# Forecasting the elimination of active trachoma: An empirical model

**DOI:** 10.1371/journal.pntd.0010563

**Published:** 2022-07-11

**Authors:** Kristen K. Renneker, Paul M. Emerson, P. J. Hooper, Jeremiah M. Ngondi

**Affiliations:** 1 International Trachoma Initiative, The Task Force for Global Health, Decatur, Georgia, United States of America; 2 RTI International, Washington DC, United States of America; The University of Hong Kong, CHINA

## Abstract

**Background:**

Great progress has been made toward the elimination of trachoma as a public-health problem. Mathematical and statistical models have been used to forecast when the program will attain the goal of the elimination of active trachoma, defined as prevalence of trachomatous inflammation—follicular in 1–9 year olds (TF_1–9_) <5%. Here we use program data to create an empirical model predicting the year of attaining global elimination of TF_1–9_.

**Methodology/Principal findings:**

We calculated the mean number of years (95% CI) observed for an implementation unit (IU) to move from a baseline TF_1–9_ prevalence ≥5% to the elimination threshold, based on the region (Ethiopia vs. non-Ethiopia) and baseline prevalence category. Ethiopia IUs had significantly different rates of reaching the TF_1–9_ elimination threshold after a trachoma impact survey (TIS) compared to non-Ethiopia IUs across all baseline categories. We used those estimates to predict when remaining active trachoma-endemic IUs (TF_1–9_ ≥5%) would have their last round of mass drug administration (MDA) based on the mean number of years required and number of MDA rounds already completed. Our model predicts that elimination of TF_1–9_ will be achieved in 2028 in Ethiopia (95% CI: 2026–2033) and 2029 outside of Ethiopia (95% CI: 2023–2034), with some IUs in East Africa predicted to be the last requiring MDA globally.

**Conclusions/Significance:**

Our empirical estimate is similar to those resulting from previous susceptible-infectious-susceptible (SIS) and mathematical models, suggesting that the forecast achievement of TF_1–9_ elimination is realistic with the caveat that although disease elimination progress can be predicted for most IUs, there is an important minority of IUs that is not declining or has not yet started trachoma elimination activities. These IUs represent an important barrier to the timely global elimination of active trachoma.

## Author summary

Mathematical and statistical models have been used to forecast the global decline of active trachoma prevalence as a result of interventions against the disease. Here we used trachoma program data to create an empirical model predicting the year of attaining global elimination. We analyzed over 20 years of trachoma implementation and survey data to calculate the mean number of rounds of mass drug administration required for implementation units to fall below the elimination threshold for active trachoma. We then compared these means against the number of rounds of mass drug administration previously undertaken for all areas still above the elimination threshold to produce a forecast of the last year of antibiotic distribution for each implementation unit. Our model predicts that elimination will be achieved in 2028 in Ethiopia (95% CI: 2026–2033) and 2029 outside of Ethiopia (95% CI: 2023–2034), with areas in East Africa predicted to be the last requiring mass drug administration globally. However, although disease-elimination progress can be predicted for most implementation units, there is an important minority of implementation units that is not declining or has not yet begun implementation. These areas represent an important barrier to the timely global elimination of active trachoma.

## Introduction

In 1996, the World Health Organization (WHO) and partners launched the WHO Alliance for the Global Elimination of Trachoma by 2020 (GET2020), which aims to reduce the prevalence of: 1) trachomatous inflammation—follicular in 1–9 year olds (TF_1–9_) to <5%, and 2) trachomatous trichiasis (TT) "unknown to the health system" of <0.2% in adults aged ≥15 years in all endemic districts worldwide. Achieving and sustaining both goals, along with the existence of a system able to identify and manage incident TT cases, evidences the elimination of trachoma as a public-health problem [1]. By June 2021, remarkable progress toward this goal had been achieved, with a reported 136 million people living in areas with a TF_1–9_ ≥5% [2], a greater than 91% reduction from 2002 estimates [3]. Furthermore, the total number of known districts above the active trachoma elimination threshold (TF_1–9_ ≥ 5%) has decreased year-on-year since a global peak in 2015 [4]. Ethiopia represents a near-majority of the global burden of active trachoma, with just under half (49.4%) of the total global population living in areas that are known to warrant interventions (antibiotics, facial cleanliness and environmental improvement) residing in Ethiopia as of June 2021 [2].

Once-yearly mass drug administration (MDA) of oral azithromycin is a key component of the WHO-recommended surgery, antibiotic, facial cleanliness, and environmental improvement (SAFE) strategy for trachoma elimination as a public-health problem (EPHP). The number of years of MDA recommended is dependent on the TF_1–9_ prevalence category of an implementation unit (IU), with a TF_1–9_ between 5–9.9% receiving one round, a TF_1–9_ between 10–29.9% receiving three rounds, a TF_1–9_ between 30–49.9% receiving five rounds, and a TF_1–9_ ≥50% receiving up to seven rounds of MDA [5–7]. After receiving these rounds of MDA, a trachoma impact survey (TIS) is conducted to determine if the elimination threshold has been achieved (TF_1–9_ <5%) and therefore inform if MDA can be stopped.

Since elimination as a public-health problem is not the same as elimination of transmission of the disease-causing organism (ocular *Chlamydia trachomatis*), there is a risk of recrudescence. In order to monitor for recrudescence, trachoma surveillance surveys (TSS) are conducted in formerly endemic IUs at least 2 years after the TF_1–9_ prevalence is demonstrated to be <5% and where there have been no additional rounds of MDA [7]. When every IU in a country has achieved both a TF_1–9_ <5% at TSS, a prevalence of TT in adults unknown to the health system <0.2%, and satisfied all other requirements, a country may submit a dossier to the WHO to be validated as having attained EPHP. By 2021, eleven countries had been validated by WHO as having achieved trachoma EPHP [3,8]. The new WHO strategic plan for NTDs, published in 2020, proposes the new target date of EPHP of trachoma in all countries by 2030 [9].

Mathematical and statistical models have been used in the trachoma community to help provide guidance on treatment strategies and forecast when EPHP may be achieved at both an IU and global level [10]. However, statistical models are not very accurate at predicting IU-level prevalence [10]. In addition, these models are based on assumptions about underlying factors related to the organism, the disease, and/or disease transmission that may not be reflected in reality, and use a small sample of data either from one setting [11,12] or only data from over 10 years ago [13]. Our forecast is different from previous models because we use actual treatment and survey data from national programs, creating IU-level predictions based on a robust global dataset. Accurate projections of stopping dates based on the past performance of similar areas could provide a useful metric for donors, country programs, and other stakeholders.

## Methods

Trachoma program data were accessed from the GET2020 database, which has data for 8,597 trachoma prevalence surveys conducted in 4,390 IUs. The GET2020 database is managed by the International Trachoma Initiative (ITI) in partnership with theWHO. The database contains data on trachoma prevalence surveys and reported SAFE implementation activities. While there is some variation in the sampling design and survey methodology for any given survey result, the majority of surveys since 2013 followed a standardized methodology to measure TF_1–9_, thanks to the implementation of the Global Trachoma Mapping Project [14] and its successor, Tropical Data [15]. An IU is defined as an administrative unit at which trachoma implementation activities take place, and typically contains 100,000–250,000 people [16]. Because of differing nomenclature and population size, we use the term "IU" to avoid confusion between different uses of the term "district," which can vary in population size from a few thousand to over a million people. Active trachoma prevalence categories, survey history, and post-2009 treatment history data are publicly available on the Global Atlas of Trachoma [17]. Countries give permission to ITI for their data to be used in support of trachoma-elimination efforts.

Survey data determined to be historic (a baseline followed by another survey in the absence of implementation) were excluded. Data from the following Pacific Islands were excluded due to atypical TF_1–9_ without presence of infection: Fiji, Papua New Guinea, Solomon Islands, and Vanuatu [18,19]. IUs that were never endemic (baseline TF_1–9_ <5%) were also excluded. Countries were grouped not by WHO region, but by geographical groupings sharing similar epidemiology.

For IUs with a TIS, a Cochran–Mantel–Haenszel test was performed to assess the relationship between region (Ethiopia vs. non-Ethiopia), baseline TF_1–9_ category, and the proportion of IUs with a TIS reaching the elimination threshold. The null hypothesis was that the proportion of IUs with a TIS above the elimination threshold was the same in Ethiopia vs. non-Ethiopia for all baseline categories.

To calculate the estimated number of rounds of MDA required to reach the elimination threshold (TF_1–9_ <5%), we took the mean of the total number of rounds of MDA required for all IUs that have reached the elimination threshold as of July 2021 based on the Ethiopia/non-Ethiopia status and baseline TF_1–9_ prevalence category. All IUs currently under the elimination threshold were included in this calculation, regardless of survey history (i.e., IUs that previously had TIS or TSS above the elimination threshold were included, as long as the most recent prevalence estimate was below the threshold). Then, for IUs with TF_1–9_ ≥5% as of July 2021, we subtracted the number of rounds already conducted in an IU since the most recent survey from the mean number of rounds projected to be required to reach the TF_1–9_ threshold in order to calculate the number of rounds remaining in each IU.


$$\begin{array}{l} Number\;of\;rounds\;remaining\;in\;an\;IU\\ \;=mean\;number\;of\;rounds\;required\\ \;-number\;of\;rounds\;already\;conducted\;in\;the\;IU\;since\;most\;recent\;survey \end{array}$$

We used the number of rounds remaining (rounded up to the nearest whole number) to project the last year of MDA required per IU, assuming one round of MDA per year. The 95% confidence intervals were calculated as ±1.96 times the standard deviation (based on the fact that 95% of the area of a normal distribution is within 1.96 standard deviations of the mean). The minimum last year of MDA was set to 2021 to account for cases where either the required number of rounds has already been reached, or where the lower bound of the 95% CI was negative and the projected last year of MDA was prior to 2021.

For cases in which there was ambiguity in the data as to whether a survey or MDA occurred first (and thus, which cycle an MDA may be part of), the month of MDA (where available) and intervention status at time of Zithromax application were used to place MDAs within a cycle. In cases where this information was not available, we assumed the MDA occurred prior to the survey, because this is more conservative for forecasting.

For the analyses using geographic groups, countries were assigned to a category according to Table 1. All analyses were done in R, using the stats and ggplot2 packages [20].

**Table 1:** Characteristics of data included.

Geographic group	Country	Number of Implementation Units included in calculation of mean number of rounds required to TF_1–9_ <5%*	Number of Implementation Units currently endemic (TF_1–9_ ≥ 5%)
Asia	Afghanistan	0	8
	Nepal	20	0
	Pakistan	9	7
	Viet Nam	22	0
	Yemen	5	25
Central Africa	Burundi	10	0
	Cameroon	22	2
	Central African Republic	5	49
	Chad	37	0
	Democratic Republic of the Congo	27	44
Eastern Africa	Egypt	0	4
	Eritrea	24	1
	Ethiopia	199	593
	Kenya	17	16
	South Sudan	0	27
	Sudan	16	14
	Tanzania	68	7
	Uganda	50	3
	Zanzibar	0	1
Latin America	Colombia	0	6
	Guatemala	2	0
	Peru	0	3
Pacific Islands	Kiribati	0	24
	Nauru	0	1
Southern Africa	Malawi	44	0
	Mozambique	35	32
	Zambia	22	23
	Zimbabwe	0	21
Western Africa	Benin	8	0
	Burkina Faso	61	0
	Côte d’Ivoire	6	45
	Gambia	15	0
	Ghana	30	0
	Guinea	19	1
	Guinea Bissau	11	1
	Mali	61	0
	Mauritania	20	0
	Morocco	5	0
	Niger	71	24
	Nigeria	100	26
	Senegal	33	0
**Total**		**1,074**	**1,008**

TF_1–9_: Trachomatous Inflammation—Follicular in children 1–9

*i.e. Implementation Units with a current TF_1–9_ <5% after implementation

## Results

A total of 2,082 IUs contributed data for 4,457 surveys, of which 2,364 were TIS. Nearly half of IUs (44%) were above the elimination threshold (TF_1–9_ ≥5%), of which the majority (60.4%) were in Ethiopia.

The proportion of IUs with a TIS result of TF_1–9_ ≥5% by Ethiopia/non-Ethiopia and baseline category is shown in Table 2. Ethiopia IUs had significantly different rates of reaching a TIS result of TF_1–9_ <5% compared to non-Ethiopia IUs across all baseline categories.

**Table 2:** Percent of IUs reaching the elimination threshold (TF_1–9_<5%) by Ethiopia/non-Ethiopia and baseline category.

	IUs ≥50% at baseline survey		IUs 30–49.9% at baseline survey		IUs 10–29.9% at baseline survey		IUs 5–9.9% at baseline survey		
Region	n with TIS	% reaching elimination threshold (TF_1–9_ <5%)	n with TIS	% reaching elimination threshold (TF_1–9_ <5%)	n with TIS	% reaching elimination threshold (TF_1–9_ <5%)	n with TIS	% reaching elimination threshold (TF_1–9_ <5%)	p-value
Ethiopia	29	10.3	179	26.3	297	40.7	68	95.6	<0.0001
Non-Ethiopia	47	44.7	173	82.1	481	85.9	47	44.7	

TIS: Trachoma Impact Survey, TF_1–9_: Trachomatous inflammation—follicular in 1–9 year olds, IU: Implementation Unit

The mean number of years required to achieve elimination by geographical and baseline TF_1–9_ prevalence category is shown in Table 3. The mean number of years to achieve elimination increases over all baseline categories (regardless of Ethiopia vs. non-Ethiopia), and was higher for IUs in Ethiopia compared to non-Ethiopia across baseline categories above 10%.

**Table 3:** Mean number of rounds (95% CI) to achieve elimination threshold (TF_1–9_ <5%) by Ethiopia/non-Ethiopia and baseline category.

Region	Baseline TF_1–9_ Category (%)	Number of IUs	Mean number of rounds of MDA (95% CI) to achieve elimination threshold (TF_1–9_ <5%)
Ethiopia	5–9.9	74	1.05 (0.63, 1.46)
Ethiopia	10–29.9	372	3.93 (−0.53, 8.4)
Ethiopia	30–49.9	318	6.74 (1.68, 11.81)
Ethiopia	≥50	29	8.67 (7.54, 9.8)
Non-Ethiopia	5–9.9	439	1.15 (−0.84, 3.13)
Non-Ethiopia	10–29.9	574	3.3 (0.25, 6.34)
Non-Ethiopia	30–49.9	182	4.25 (−0.75, 9.24)
Non-Ethiopia	≥50	60	7.14 (1.39, 12.9)

TF_1–9_: Trachomatous inflammation—follicular in 1–9 year olds, IU: Implementation Unit, MDA: Mass Drug Administration

**Fig 1:**
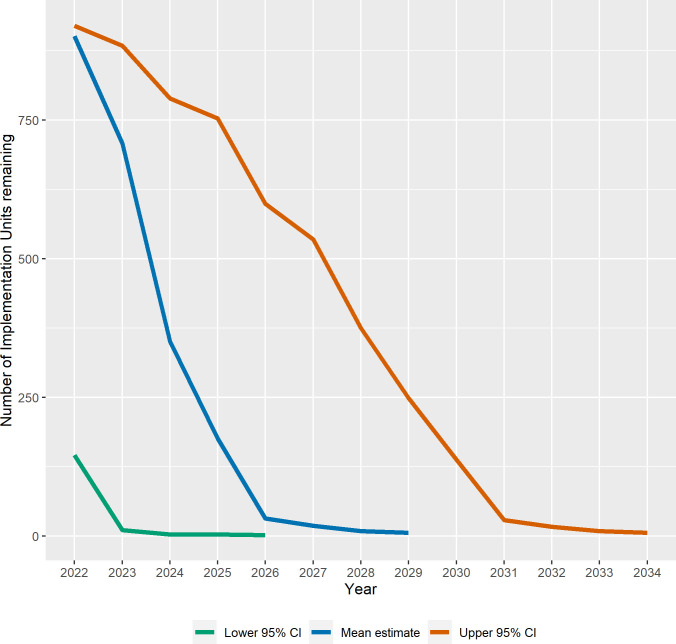
The mean, lower 95% CI, and upper 95% CI forecast number of Implementation Units remaining requiring Mass Drug Administration by year.

**Fig 2:**
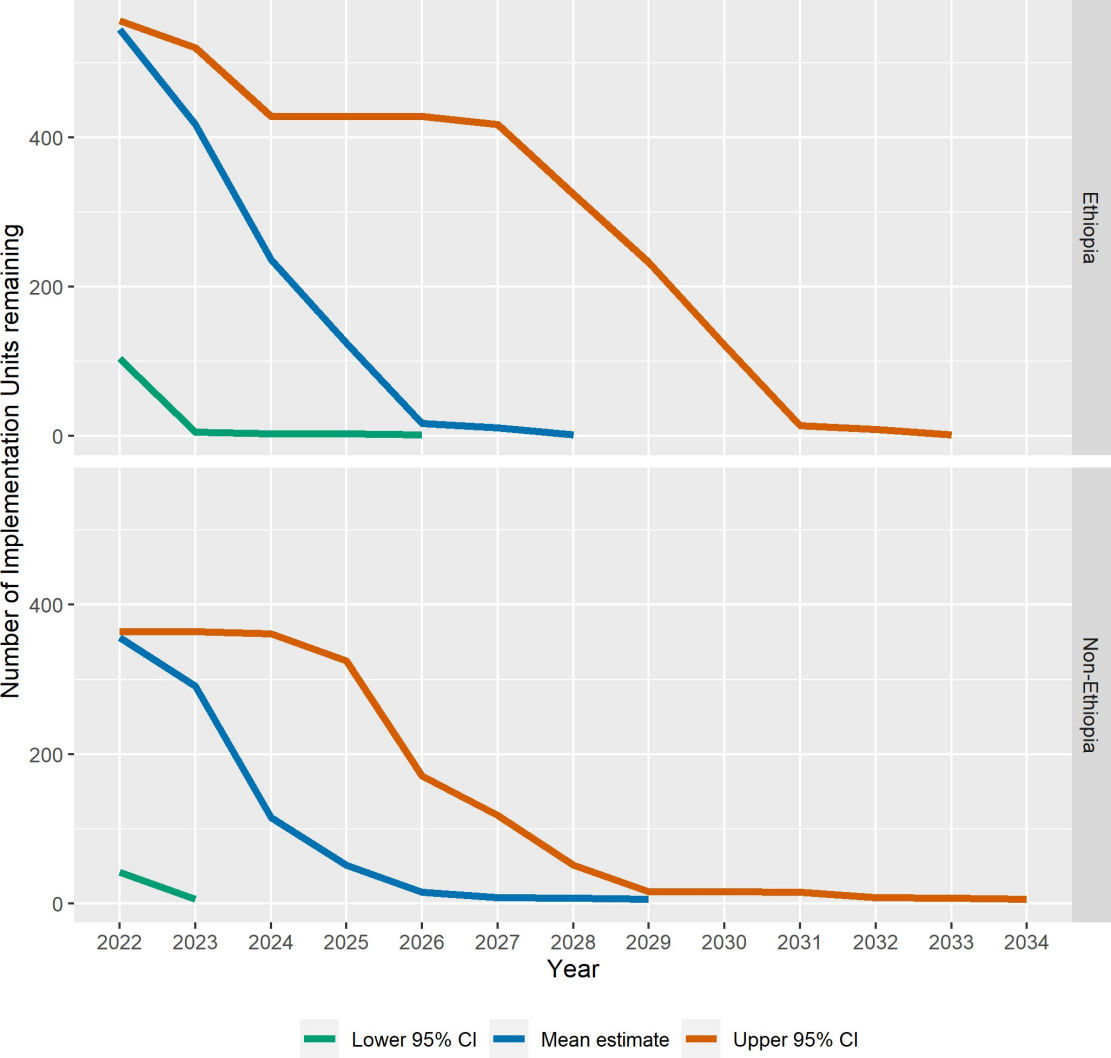
Mean, lower 95% CI, and upper 95% CI forecast number of Implementation Units remaining requiring Mass Drug Administration by year, Ethiopia vs. non-Ethiopia.

**Fig 3:**
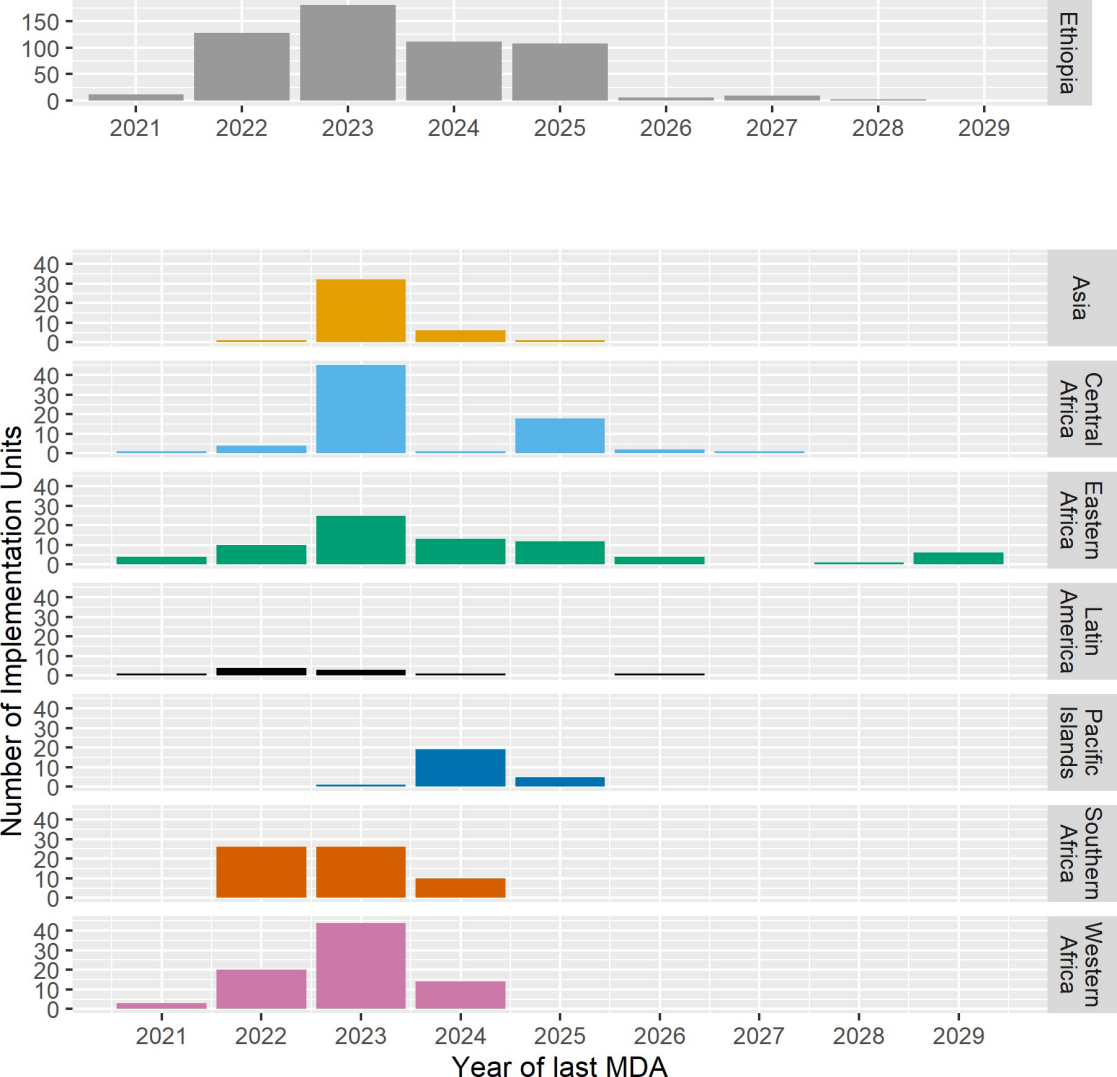
The number of Implementation Units with Mass Drug Administration (MDA) by year of last MDA and geographic group. Note the different vertical axis for Ethiopia compared to the other geographic groups.

**Fig 4:**
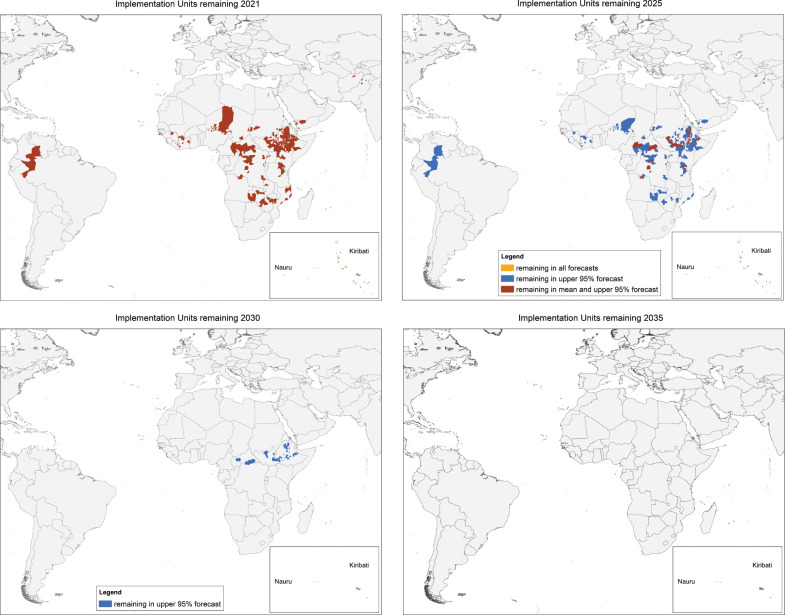
Maps showing the Implementation Units (IUs) known to remain requiring Mass Drug Administration (MDA) in 2021 and the mean, upper 95% CI, and lower 95% CI IUs forecast to remain requiring MDA in 2025, 2030, and 2035. Basemap provided by the International Trachoma Initiative.

Forecast progress in the program is shown in three ways: a) as the number of currently known endemic IUs remaining by year (Figs 1 and 2); b) the forecast mean last year of MDA before achieving TF_1–9_ <5% by geographic group (Fig 3), and c) as maps showing IUs currently requiring MDA and those forecast to be remaining in the program in 5-year intervals (Fig 4).

The mean and 95% upper and lower confidence estimates for the forecast number of IUs remaining by year for the global program are shown in Fig 1. For each year, the number of remaining MDAs are expected to occur during that year; that is, by the end of a given year, those IUs will have attained TF_1–9_ <5% and will no longer require MDA. The forecast mean last year of MDA for the global program is 2029 (95% CI: 2026–2034). Using the mean forecast, we anticipate that more than 50% of IUs currently above the elimination threshold will achieve elimination by 2024, with over 90% of the currently endemic IUs moving below the TF_1–9_ elimination threshold by 2026 (Fig 1).

Because Ethiopia represents the majority of IUs still above the elimination threshold, and Ethiopian IUs have significantly lower rates of achieving TF_1–9_ <5% at TIS (Table 2), we forecast the last year of MDA separately for Ethiopia vs. non-Ethiopia. The mean and 95% upper and lower confidence estimates for the forecast number of IUs remaining by year for Ethiopia vs. non-Ethiopia are shown in Fig 2. Our model predicts that elimination will be achieved in 2028 in Ethiopia (95% CI: 2026–2033) and 2029 outside of Ethiopia (95% CI: 2023–2034).

The mean last year of MDA differs by geographic group (Fig 3). The East Africa region (not including Ethiopia) is forecast to have the last remaining IUs requiring MDA, with a last year of MDA of 2029. The number of IUs with each last forecast year of MDA in each geographic group is shown.

Fig 4 shows the mean, upper, and lower forecasts of IUs remaining geographically by year: IUs currently above threshold (2021), IUs remaining in 2025, 2030, and finally 2035, when the upper estimate forecasts no IUs remaining.

## Discussion

Our model produced similar results to the previously reported models [11]: significant progress in the global program is expected by 2025, with a chance of attaining global elimination of active trachoma by 2029. This similarity lends validity to the results of both our and others’ forecasts. Our model differs from previous models in two key aspects. Although our model is very simplistic and built on a number of assumptions, it is based on observational data of what has happened and projects that out to what may happen in the future; in contrast, previous models that fit the data well accurately predict the past, but do not provide a forecast for the future [13,21–24]. Our model also differs from others in how it handles random variation; because we use empirical data, variation observed in the data is already built into our model. This contrasts the mathematical models, which must add an explicit stochastic element in order to achieve a good fit.

In each baseline/Ethiopia vs. non-Ethiopia category, the mean number of rounds required to achieve elimination is higher than the WHO program guideline number of rounds (except for non-Ethiopia IUs with a baseline between 30–49.9%, which require 4.25 rounds on average, meaning some are able to stop MDA before the 5 rounds recommended by program guidelines; this is likely due to a number of IUs in this category opting to conduct a TIS after 3 rounds of MDA, rather than the 5 years specified by program guidelines). Programs should keep this in mind when allocating resources and planning realistic implementation timelines.

Our forecast relies on several assumptions. We recognize that some of these assumptions, such as that there will be no interruptions in SAFE programming, are optimistic. Additionally, in accordance with current WHO guidelines, once IUs have reached the elimination threshold, MDA stops and the IU moves into the surveillance phase, with any necessary surgical interventions, facial cleanliness and environmental improvements continuing. After at least two years without antibiotic pressure, a TSS is conducted to determine whether TF_1–9_ <5% has been sustained in the absence of the mass distribution of antibiotics. Data suggest that around 9% of IUs show an increase in TF_1–9_ above the threshold at their TSS [25] and must resume mass antibiotic distribution. Based on our global survey data, it is reasonable to expect that IUs with a higher baseline prevalence have a greater frequency of requiring additional MDA following a surveillance survey than IUs with low baseline prevalence [17]. Therefore, our model assumes that the last year of MDA actually corresponds with the end of antibiotic distribution, but this is unlikely to be the case in all IUs. Furthermore, once all IUs in a country sustain the elimination threshold at a TSS, the national program writes and submits an elimination dossier to WHO for validation of EPHP. Thus, this forecast only shows when MDA will stop in all IUs in a country; validation can reasonably be expected at least three years later.

Additionally, this model assumes that there will be no technological or methodological advances in programming that accelerate achievement of the TF_1–9_ <5%. Operational research and clinical trials have demonstrated that more-frequent-than-annual MDA may accelerate progress to elimination [21–24]. A WHO Informal Consultation held in December 2021 recommended more intensive implementation in areas with persistent or recrudescent infection. There is ongoing research, including the Stronger SAFE trial [26] in Ethiopia, working to identify ways to speed progress to the elimination target through enhanced vector control, hygiene promotion, sanitation, and more-frequent-than-annual MDA. The findings of these trials are increasingly important in the elimination endgame.

The COVID-19 pandemic affected the provision of health services worldwide, including MDA. Modelling work has indicated that, of the preventative chemotherapy NTDs, trachoma was one of the diseases most likely to "bounce back" the quickest, especially in high-transmission areas, and therefore catch-up MDA strategies in these settings are recommended, with such strategies having the potential to accelerate the timeline to elimination [27]. In 2022, MDA activities can be expected to resume at scale as country programs adopt WHO guidelines [28].

Another assumption that we make is that there will be sufficient resources available (donor funds, drug donation, etc.) to implement SAFE and sustain elimination, which requires multi-sectoral partnerships and continued resource allocation before, during, and after achievement of elimination goals [29]. However, one resource that we cannot increase is time. While our focus is often on Ethiopia, which has over 50% of remaining endemic IUs, there are still highly endemic IUs in other countries where interventions have not yet started, and these areas account for the fact that Ethiopia is forecast to finish MDA prior to non-Ethiopia. Our model indicates that South Sudan will be the last country to reach the trachoma EPHP targets because of many stalled attempts to start in each IU since the program was initiated in the early 2000s. For every year that we do not start interventions, the year of attaining global elimination is pushed further out. Our model does not include areas that are suspected endemic; there are currently 187 districts [30] that are suspected endemic and may require baseline mapping to determine TF_1–9_ prevalence. Based on recent mapping results [17], the vast majority of these remaining un-surveyed districts will be found to be non-endemic and not require intervention.

Several of the Pacific Islands (Fiji, Papua New Guinea, Solomon Islands, Vanuatu) are excluded from this analysis. Based on operational research [19] and following recommendations from the WHO Regional Office of the Western Pacific [18], these countries are not following the pattern for trachoma as a public-health problem and are no longer planning to conduct MDA.

### Limitations

An inherent limitation of all models is that they assume that the future will behave like the past. In order to be included in our calculation of the mean number of rounds of MDA required to reach the active trachoma elimination threshold, IUs have to have already reached TF_1–9_ <5% at an impact survey; therefore, it is difficult to predict the timeline to elimination for IUs that are behaving differently (i.e., that have not yet reached TF_1–9_ <5% at a TIS), since treatment is still underway and we do not yet know how many additional rounds of MDA will be needed. Some IUs have had 10 rounds of MDA or more and still have not yet reached TF_1–9_ <5% at TIS [17]. Although these IUs are few in number, they will have considerable impact on the overall forecast year of attaining global elimination, since every IU must reach TF_1–9_ <5% to stop MDA and sustain that progress for >2 years. Over time, these outliers will become increasingly important in determining global program progress. In these IUs, elimination remains more a question of "if" than "when"; thus research into risk factors for a TIS or TSS TF_1–9_ ≥5% and novel ways to address the disease in these areas will be a vital component of the global trachoma elimination strategy.

## Conclusions

We can be reasonably certain that in the coming years (2022–2026) there will be a great deal of progress towards elimination of active trachoma, with over 50% of IUs forecast to achieve the TF_1–9_ elimination threshold between now and 2024 and an additional 40% estimated to reach this threshold by 2026. However, because global elimination requires sustained elimination in 100% of IUs, we have less confidence in our prediction of how much longer those trailing IUs will take to achieve TF_1–9_ elimination, which is why we need to start work now to make progress in those areas. In a global program, we must work everywhere, so by starting interventions where possible and exploring alternative treatment strategies that may accelerate progress in areas where intervention is not currently possible or where standard intervention does not appear to be working, we can achieve the goal of global elimination of trachoma.

### Disclaimer

The views expressed in this article are the views of the authors alone and do not necessarily reflect the decisions, policies or views of the institutions with which they are affiliated, or of the funding agencies.
